# Editorial: Advanced therapeutic delivery for the management of chronic respiratory diseases

**DOI:** 10.3389/fmed.2022.983583

**Published:** 2022-08-09

**Authors:** Keshav Raj Paudel, Dinesh Kumar Chellappan, Ronan MacLoughlin, Terezinha de Jesus Andreoli Pinto, Kamal Dua, Philip M. Hansbro

**Affiliations:** ^1^Centre for Inflammation, Centenary Institute and University of Technology Sydney, Faculty of Science, School of Life Sciences, Sydney, NSW, Australia; ^2^Department of Life Sciences, School of Pharmacy, International Medical University, Kuala Lumpur, SGR, Malaysia; ^3^Research and Development, Science and Emerging Technologies, Aerogen Limited, Galway Business Park, Galway, Ireland; ^4^School of Pharmacy and Biomolecular Sciences, Royal College of Surgeons in Ireland, Dublin, Ireland; ^5^School of Pharmacy and Pharmaceutical Sciences, Trinity College, Dublin, Ireland; ^6^Department of Pharmacy, Faculty of Pharmaceutical Sciences, University of São Paulo, São Paulo, Brazil; ^7^Discipline of Pharmacy, Graduate School of Health, University of Technology Sydney, Sydney, NSW, Australia; ^8^Faculty of Health, Australian Research Centre in Complementary and Integrative Medicine, University of Technology Sydney, Ultimo, NSW, Australia

**Keywords:** drug delivery, chronic respiratory disease, pulmonary medicine, therapeutic delivery, drug discovery

The management of chronic respiratory/lung diseases (CRDs), utilizing outdated conventional therapeutic models has urged the need for drug repurposing, and has renewed the interest in discovery of novel, advanced therapeutic approaches. Novel advanced drug delivery systems are lately emerging with a versatility to manage CRDs (1, 2). Contemporary scientists have become more inclined toward exploring the application of nanoparticle-based formulations (3, 4) or genetic materials such as siRNA, miRNA, and decoy oligonucleotide-targeted/loaded delivery system (5, 6) to effectively manage inflammatory lung diseases or to delay the exacerbation of CRDs. Interestingly, a considerable number of drug-loaded nanocarriers have proven to be effective in *in vitro* and *in vivo* studies (7–9). There has been a lack of robust therapeutic delivery methods for the management of CRDs. Conventional oral drug delivery approaches are associated with low systemic bioavailability, and deterioration in the gastrointestinal sites. Therefore, higher doses are required to attain therapeutic efficacy which might result in multiple unwanted/off-target effects (1). Advanced therapeutic delivery systems may be employed to overcome these risks by enhancing the potency of therapeutics with comparatively less dose and fewer off-target effects than conventional approaches. Therefore, more scientific studies are essential to further validate the implementation of different advanced drug delivery systems for CRDs.

In our special issue, we had invited submissions ranging from original research, brief reports, and review (narrative/systematic) articles that fall within the scope of our theme, “Advanced Therapeutic Delivery for the Management of Chronic Respiratory Diseases.” A total of ten articles were published on this topic, including six original research articles, one brief research report, and three systematic review articles.

A meta-analysis by Huang, Pei et al., reported that the bacterial lysates could be beneficial for COPD patients. Twenty studies were pooled in this meta-analysis where, the data suggests that the lysates were efficacious to alleviate symptoms, with decreased exacerbation rate and mean number of exacerbations with acceptable side effects.

Another meta-analysis by Yu et al., suggested that proton pump inhibitor treatment could decrease the case fatality rate in COPD, occurrence of gastrointestinal hemorrhage, and number of acute exacerbations. As the study was limited to China, the authors suggested the need for large-scale randomized control trials to further validate their findings and avoid high risk of bias.

Kim et al., highlighted that the use of statin was not correlated with the incidence of COPD in adults. Nevertheless, it was linked with a decreased likelihood of exacerbations in COPD participants, with a greater risk reduction with lipophilic statin use.

Hong et al., studied the variations in intensive care unit outcomes corresponding to the chemotherapy type administered to patients with lung cancer (LC). It was observed that the targeted chemotherapy may contribute to increasing access to critical care for LC and improved critical care outcomes of LC patients.

Lai et al., reported on clinical and inflammatory features of the AtyPical Asthma in China (APAC) cough variant asthma cohort. Cough variant asthma was found to be distinctive from classic asthma with regards to clinical features, lung function, and airway inflammation. Quality of life in APAC cohort was poorly diminished despite better asthma control scores.

Huang, Fu et al., investigated the predictors of a minimal clinically important difference following omalizumab treatment in adult patients with severe allergic asthma (SAA). Interestingly, no predictor of lung function or asthma control was found. The findings suggest that their study might be helpful when choosing treatment for adult patients with SAA to benefit the most from omalizumab treatment.

A brief research report by Li et al., explored the potential of transpedal lymphangiography procedure using a high dose (>20 ml) ethiodized oil in the management of postoperative chylothorax. It was observed that high dose ethiodized oil was feasible, safe, and effective model for the management of high-flow (>1,000 ml/day) postoperative chylothorax.

Liu et al., studied the application of adjusted single-breath helium dilution (SBHD) for the measurement of total lung volume in the patients with obstructive lung disease (OLD). The SBHD approach was correlated with whole-body plethysmography to measure the total lung volume. However, SBHD method presents limitations in determining the total lung volume in patients with OLD. The authors established SBHF as a functional and reliable correction equation to precisely examine the total lung volume of patients with OLD.

A systematic review and meta-analysis by Lu et al., highlighted the effectiveness of telemonitoring (TM) to reduce the COPD exacerbation occurrence in patients with past exacerbation history. It was found that TM can decrease patient visits to the emergency room, exacerbation-related readmissions, acute exacerbation-related hospital stay, mortality, and the St. George's respiratory questionnaire score. This study suggested that execution of TM could be a promising strategy that could ease the long-term management of acute exacerbation COPD.

Ju et al., investigated the epidemiology and prognosis of invasive fungal disease (IFD) in Chinese lung transplant recipients (LTRs). The most prevalent pathogens were *Aspergillus* (57.3%), *Candida* (19.5%), and *Pneumocystis jiroveci* (13.4%). With the multivariate logistic regression analysis, it was revealed that anastomotic disease, cytomegalovirus (CMV) pneumonia, and pre-transplantation IFD were linked with increased odds of IFD, while double-lung transplantation was linked with decreased odds of IFD. The anastomotic disease was linked with increased odds of death and that *Pneumocystis jiroveci* pneumonia prophylaxis was linked with decreased odds of death. This study concluded that IFD is prevalent among LTRs in Southern China, with *Aspergillus* the most common pathogen suggesting optimization of prophylaxis based on likely pathogens.

In conclusion, this topical collection has provided some new experimental data and updated reviews about the advanced therapeutic delivery for the management of chronic respiratory diseases.

## Author contributions

All authors listed have made a substantial, direct, and intellectual contribution to the work and approved it for publication.

## Conflict of interest

The authors declare that the research was conducted in the absence of any commercial or financial relationships that could be construed as a potential conflict of interest.

## Publisher's note

All claims expressed in this article are solely those of the authors and do not necessarily represent those of their affiliated organizations, or those of the publisher, the editors and the reviewers. Any product that may be evaluated in this article, or claim that may be made by its manufacturer, is not guaranteed or endorsed by the publisher.
